# Supplementary material for: Curiosity or savouring? Information seeking is modulated by both uncertainty and valence
Source: PLoS One. 2021 Sep 24;16(9):e0257011. doi: 10.1371/journal.pone.0257011 (PMC8462690; doi:10.1371/journal.pone.0257011)
Supplement: S1 Text — (DOCX) [file pone.0257011.s001.docx]

**S1 Text: Analyses of Experiment 1A, 1B, 1C and 2 using repeated measures ANOVAs**

­­­

**Repeated Measures ANOVAs in SPSS**

In addition to the analyses reported in the main text, we performed similar analyses using repeated measures ANOVAs in SPSS (RRID:SCR_002865). These analyses were performed to accommodate readers that are used to interpreting frequentist statistics instead of Bayesian credible intervals and to verify that our conclusions do not depend on the analytical framework employed. The conclusions derived from the repeated measures ANOVAs are essentially the same as the conclusions reported in the main text (see S1 Table).

To this end, we divided the values of outcome uncertainty into “low outcome uncertainty” and “high outcome uncertainty”, such that approximately 50% of the trials were indicated as being low outcome uncertainty (outcome uncertainty <= 350 in Experiment 1A and outcome uncertainty <= 200 in Experiment 1B, 1C and 2) and approximately 50% as high outcome uncertainty (outcome uncertainty > 350 in Experiment 1A and outcome uncertainty > 200 in Experiment 1B, 1C and 2). Additionally, we divided the values of absolute expected value into “low expected value” (expected value (absolute) < 50) and “high expected value” (expected value (absolute) > 50). Note that the trials with absolute expected value = 50 were omitted from the analyses. This was done because the values of absolute expected value are perfectly centered around expected value (absolute) = 50, precluding us to classify these trials as being either low or high expected value.

We performed a 2 (outcome valence: gain, loss) x 2 (outcome uncertainty: low, high) x 2 (expected value (absolute): low, high) repeated measures ANOVA with outcome valence (gain/loss), outcome uncertainty (low/high) and absolute expected value (low/high) as within-subject factors. The dependent variable was either mean curiosity as indicated by means of the curiosity ratings (Experiment 1A, 1B and 1C) or mean percentage willingness to wait, as indicated by participants’ willingness to wait decisions (Experiment 2). If the interaction effects between “outcome valence (gain/loss)” and “outcome uncertainty” or between “outcome valence (gain/loss)” and “expected value (absolute)” were significant, we ran 2 (outcome uncertainty: low, high) x 2 (expected value (absolute): low, high) repeated measures ANOVAs on the gain and loss trials separately. This allowed us to assess the significance of outcome uncertainty and absolute expected value on curiosity or willingness to wait for the gain and loss trials separately.

**Bayesian Repeated Measures ANOVAs in JASP**

In JASP (RRID:SCR_015823), we performed the Bayesian equivalent of the repeated measures ANOVAs reported above. We used the default Cauchy prior to compute Bayes Factors for each effect. For interpretability of analyses with multiple factors, we used model averaging across matched models to get a single BF for each effect in the repeated measures ANOVA. Specifically, each BF is computed as the sum of P(model|data) of all models containing the effect of interest (but no interactions with the effect of interest), divided by the sum of P(model|data) of all the models that are stripped of this effect of interest. As such, each Bayes Factor reflects the change from prior to posterior inclusion odds and can intuitively be understood as the amount of evidence that the data gives for including an experimental factor in a model (BFincl). The BFincl will converge to zero when the factor should not be included in the model, or to infinity when the factor should be included in the model. Values close to one indicate that there is not enough evidence for either conclusion.

**Results Experiment 1A, 1B and 1C**

First of all, curiosity strongly increased with outcome uncertainty (**Exp. 1A** RMA: F(1,33) = 51.7, *p* = 3.07e-8, η_p_^2^ = .61, BF = 1.54e+20; **Exp. 1B** RMA: F(1,32) = 57.5, *p* = 1.22e-8, η_p_^2^  = .64, BF = 1.94e+19; **Exp. 1C** RMA: F(1,32) = 135.6, *p* = 4.80e-13, η_p_^2^  = .81, BF = 5.43e+48), such that participants were more curious about high (**Exp. 1A** *M* = 2.79; *SD* = .43; **Exp. 1B** *M* = 3.04; *SD* = 0.44; **Exp. 1C** *M* = 3.17; *SD* = .39) compared with low outcome uncertainty (**Exp. 1A** *M* = 2.21; *SD* = .48; **Exp 1B** *M* = 2.24; *SD* = 0.40; **Exp 1C** *M* = 2.16; *SD* = .37) in Experiment 1A, 1B and 1C (Table 1). There was also a main effect of outcome valence, such that curiosity was higher for gains (**Exp. 1A** *M* = 2.69; *SD* = .44; **Exp. 1B** *M* = 2.99; *SD* = 0.51; **Exp. 1C** *M* = 2.82; *SD* = .38) compared with losses (**Exp. 1A** *M* = 2.43; *SD* = .48; RMA: F(1,33) = 16.4, *p* = 2.93e-4, η_p_^2^  = .33, BF = 1.27e+6; **Exp. 1B** *M* = 2.29; *SD* = 0.58; RMA: F(1,32) = 19.8*, p* = 9.69e-5, η_p_^2^  = .38, BF = 3.07e+15; **Exp. 1C** *M* = 2.49; *SD* = .37; RMA: F(1,32) = 16.7*, p* = 2.75e-4, η_p_^2^  = .34, BF = 5.69e+7). Crucially, there was no interaction between outcome uncertainty and outcome valence when analyzing the data with a repeated measures ANOVA for all experiments (**Exp. 1A** RMA: F(1,33) = 1.7, *p* = .20, η_p_^2^  = .049, BF = .21; **Exp 1B** RMA: F(1,32) = 3.6, *p* = .066, η_p_^2^  = .10, BF = .21; **Exp 1C** RMA: F(1,32) = 4.07e-5*, p* = .995, η_p_^2^  = 1.27e-6, BF = .20).

There was no significant interaction between absolute expected value and outcome valence in Experiment 1A (**Exp. 1A** RMA: F(1,33) = .38, *p* = .54, η_p_^2^  = .011, BF = .20). Also, curiosity did not increase with absolute expected value in Experiment 1A (**Exp. 1A** RMA: F(1,33) = 2.4, *p* = .13, η_p_^2^  = .068, BF = .44), indicating that curiosity did not scale with reward magnitude. In other words, there was no difference in curiosity ratings for low gains (*M* = 2.59; *SD* = 0.44) compared with high gains (*M* = 2.69; *SD* = 0.56) and no difference in curiosity ratings for low losses (*M* = 2.35; *SD* = 0.47) compared with high losses (*M* = 2.40; *SD* = 0.56). In Experiment 1B and 1C, however, there was a significant main effect of absolute expected value, such that curiosity was higher for high compared with low absolute expected value (**Exp. 1B** RMA: F(1,32) = 32.8, *p* = 2.42e-6, η_p_^2^  = .51, BF = 1.59; **Exp. 1C** RMA: F(1,32) = 26.3, *p* = 1.39e-5, η_p_^2^  = .45, BF = 862.7). In Experiment 1B, there was also evidence for an interaction between absolute expected value and outcome valence (**Exp. 1B** RMA: F(1,32) = 12.4, *p* = .001, η_p_^2^  = .28, BF = .51), indicating that the effects of absolute expected value differ between gain and loss trials. Indeed, analyses of the gain trials and loss trials separately, revealed a positive relationship between absolute expected value and curiosity in the gain trials, such that participants were more curious for higher (*M* = 3.10; *SD* = 0.56) compared with lower gains (*M* = 2.82; *SD* = 0.50; **Exp 1B** RMA: F(1,32) =46.72, *p* = 9.91e-8, η_p_^2^  = .59, BF = 412.3). However, there was no relationship between absolute expected value and curiosity in the loss trials (**Exp. 1B** RMA: F(1,32) =1.8, *p* = .194, η_p_^2^  = .052, BF = .25), such that there was no difference in curiosity about higher losses (*M* = 2.29; S*D* = 0.64) compared with lower losses (*M* = 2.23; *SD* = 0.50).

In Experiment 1C, there was no interaction between outcome valence and absolute expected value (**Exp. 1C** RMA: F(1,32) = 2.5, *p* = .12, η_p_^2^  = .074, BF = .23), indicating that participants were more curious about higher compared with lower gains and losses.

**Results Experiment 2**

In Experiment 2, we tested people’s curiosity more implicitly by means of assessing their willingness to wait to see the outcome (Table 2). Consistent with Experiment 1A, 1B and 1C, willingness to wait increased with outcome uncertainty (**RMA:** F(1,33) = 63.6, *p* = 3.38e-9, η_p_^2^  = .66, BF = 5.32e+31), such that participants were willing to wait in a higher percentage of trials with high (*M* = 63.6; *SD* = 20.5) compared with low (*M* = 25.4; *SD* = 19.5) outcome uncertainty. Also, willingness to wait was higher for gain (*M* = 55.1; *SD* = 23.6) compared with loss trials (*M* = 35.6; *SD* = 14.5; **RMA:** *F*(1,33) =18.4, *p* = 1.46e-4, η_p_^2^  = .36, BF = 3.27e+8). The interaction between outcome uncertainty and outcome valence on willingness to wait did not reach significance (**RMA:** *F*(1,33) =2.9, *p* = .099, η_p_^2^  = .080, BF = .24).

There was a significant interaction between absolute expected value and outcome valence (**RMA:** *F*(1,33) =16.0, *p* = 3.36e-4, η_p_^2^  = .33, BF = 1.07), indicating that the effects of absolute expected value differed between gain and loss trials. Indeed, analyses of gain and loss trials separately revealed a positive relationship between absolute expected value and willingness to wait in the gain trials (**RMA:** *F*(1,33) = 5.9, *p* = .021, η_p_^2^  = .15, BF = .61), such that participants were more willing to wait for higher gains (*M* = 54.4; *SD* = 25.0) than for lower gains (*M* = 49.1; *SD* = 23.8). However, there was a negative relationship between absolute expected value and willingness to wait in the loss trials (**RMA:** *F*(1,33) = 9.1, *p* = .005, η_p_^2^  = .22, BF = .64), such that participants were *less* willing to wait for higher losses (*M* = 31.4; *SD* = 13.7) compared with lower losses (*M* = 36.5; *SD* = 14.6). There was no main effect of absolute expected value on willingness to wait (**RMA:** *F*(1,33) =.003, *p* = .96, η_p_^2^  = 7.85e-5, BF = .14).

**Discussion**

The repeated measure ANOVAs largely replicated the findings using linear mixed modeling reported in the main text.
