# Supplementary material for: Curiosity or savouring? Information seeking is modulated by both uncertainty and valence
Source: PLoS One. 2021 Sep 24;16(9):e0257011. doi: 10.1371/journal.pone.0257011 (PMC8462690; doi:10.1371/journal.pone.0257011)
Supplement: S2 Text — (DOCX) [file pone.0257011.s002.docx]

**S2 Text: Including the mixed outcome valence condition**

We performed additional analyses on the data of Experiment 1A that were not of primary interest, but relevant to understand the data. All experiments reported in the manuscript included gain trials (in which both monetary values are positive) and loss trials (in which both monetary values are negative). However, Experiment 1A also included mixed trials, in which one monetary value is positive and one monetary value is negative. In these trials, there is additional uncertainty about the valence of the outcome (i.e. about whether people would actually gain or lose money). We aimed to investigate whether curiosity would be enhanced with this added uncertainty. To this end, we ran a model in which we compared the mixed trials with the gain trials and a model in which we compared the mixed trials with the loss trials.

**Statistical analyses including mixed trials**

To compare the mixed trials with the gain and the loss trials, we modeled the data using the brm function of the BRMS package (1) in a similar way as described before. In the first model we compared the gain trials with the mixed trials. To this end, “curiosity rating (1 - 4)” was included as ordinal dependent variable and the main effects of “outcome valence (gain/mixed)”, “outcome uncertainty” and “expected value” were included as fixed effects as well as the interaction effects between “outcome valence (gain/mixed)” and “outcome uncertainty” and between “outcome valence (gain/mixed)” and “expected value”. The model included a full random effects structure (2,3) meaning that a random intercept and random slopes for all effects were included per participant. In the second model, we compared the loss trials with the mixed trials. To this end, we modeled the data in a similar way as described above, except that the gain trials were replaced with the loss trials.

The main difference between these analyses and the primary statistical analyses (*see main text – 2. Methods – 2.4 Experimental Design & Primary Statistical Analyses*), was the way in which the values for expected value were included in the analyses. Since expected value was always positive in the gain context, always negative in the loss context, and could be positive or negative in the mixed context, the exact expected values differed between the three contexts. In order to account for this, we standardized the values of expected value for the three contexts separately and we used these values in the models. It should be noted that the expected values in the gain context range from less to more positive values (from gaining less money to gaining more money) in the loss context from more negative to less negative values (from losing more money to losing less money) and in the mixed context from negative values to positive values (from losing money to gaining money).

All other conventions regarding the modeling are as for the primary statistical analyses (see *main text – 2. methods* – *2.4 Experimental Design & Primary Statistical Analyses*).

Additionally, we performed similar analyses using repeated measures ANOVAs in SPSS and the Bayesian equivalent of the repeated measures ANOVAs in JASP. To this end, we divided the values of outcome uncertainty into “low outcome uncertainty” and “high outcome uncertainty”, such that approximately 50% of the trials were indicated as being low outcome uncertainty (outcome uncertainty <= 350) and approximately 50% as high outcome uncertainty (outcome uncertainty > 350). Additionally, we divided the values of expected value into “low expected value” and “high expected value” for the contexts separately. For the gain context this would mean that “low expected value” < 50 and “high expected value” > 50, for the loss context, “low expected value” < -50 and “high expected value” > -50 and for the mixed context “low expected value”< 0 and “high expected value” > 0. Note again that the values of expected value are perfectly centered around expected value = 50 in the gain context, expected value = -50 in the loss context and expected value = 0 in the mixed context. This precludes us to classify the trials with these values of expected value as either low or high expected value and therefore we omitted these trials from the analyses.

We performed a 2 (outcome valence: gain, mixed) x 2 (outcome uncertainty: low, high) x 2 (expected value: low, high) repeated measures ANOVA with outcome valence (gain/mixed), outcome uncertainty and expected value as within-subject factors. The dependent variable was mean curiosity, as indicated by participants’ curiosity ratings. If the interaction effects between “outcome valence (gain/mixed)” and “outcome uncertainty” or between “outcome valence (gain/mixed)” and “expected value” were significant, we ran a 2 (outcome uncertainty: low, high) x 2 (expected value (absolute): low, high) repeated measures ANOVA on the gain and mixed trials separately. This allowed us to assess the significance of outcome uncertainty and expected value (absolute) on willingness to wait of the gain and mixed trials separately. To compare the mixed trials to the loss trials in the same way, we performed the same repeated measures ANOVAs with the loss instead of the gain trials.

**Data visualization**

The data visualization was performed in the same way as described in the main text (with addition of a mixed context; see Supplementary Fig 3).

**Results**

When comparing the mixed trials with the gain trials (S3 Fig), we found that participants were more curious about the outcome of gain trials (*M* = 2.69; *SD* = 0.44) compared with mixed trials (*M* = 2.38; *SD* = 0.39; **BRMS:** 95% CI [.23,.55]; **RMA:**  *F*(1,33) = 25.2, *p* = 1.74e-5, η_p_^2^  = .43, BF = 5.28e+6). We again found that curiosity increased with outcome uncertainty (**BRMS:** 95% CI [.67,1.19]; **RMA:**  *F*(1,33) = 51.7, *p* = 3.05e-8, η_p_^2^  = .61, BF = 1.20e+24), but not with expected value (**BRMS:** 95% CI [-.05,.21]),; **RMA:**  *F*(1,33) = .76, *p* = .39, η_p_^2^  = .023, BF = .18). Additionally, there was an interaction between outcome uncertainty and gain vs. mixed trials (**BRMS:** 95% CI [-.14,-.01]); **RMA:**  *F*(1,33) = 5.5, *p* = .025, η_p_^2^  = .14, BF = .41), such that the effect of outcome uncertainty was stronger for mixed trials compared with gain trials. There was no interaction between expected value and gain vs. mixed trials (**BRMS:** 95% CI [-.06,.17]); **RMA:**  *F*(1,33) = 2.05, *p* = .16, η_p_^2^  = .058, BF = .33).

When comparing the mixed trials with the loss trials (S3 Fig), we found no difference between curiosity about the outcome for loss trials (*M* = 2.43; *SD* = .48) and mixed trials (*M* = 2.38; *SD* = 0.39; **BRMS:** 95% CI [-.12,.22]; **RMA:**  *F*(1,33) = .20, *p* = .66, η_p_^2^  = 5.87e-3, BF = .16), indicating that the mixed trials were evaluated in a similar way as the loss trials. We again found that curiosity increased with outcome uncertainty (**BRMS:** 95% CI [.64,1.16]; **RMA:**  *F*(1,33) = 47.1, *p* = 7.84e-8, η_p_^2^  = .59, BF = 4.54e+22), but not with expected value (**BRMS:** 95% CI [-.16,.09]); **RMA:** *F*(1,33) = .61, *p* = .44, η_p_^2^  = .018, BF = .17). Additionally, there was an interaction between outcome uncertainty and loss vs. mixed trials (**BRMS:** 95% CI [-.17,-.03]); **RMA:**  *F*(1,33) = 10.2, *p* = .003, η_p_^2^  = .24, BF = .79), such that the effect of outcome uncertainty was stronger for mixed trials compared with loss trials. There was no interaction between expected value and loss vs. mixed trials (**BRMS:** 95% CI [-.15,.02]); **RMA:**  *F*(1,33) = .32, *p* = .58, η_p_^2^  = .010, BF = .19).

**Discussion**

The mixed trials allowed us to investigate whether curiosity would be enhanced when there is additional uncertainty about the valence of the outcome (i.e. when there was uncertainty regarding whether people would actually gain or lose money). The results show that curiosity was higher for gain trials than for mixed trials, but there was no difference in curiosity between mixed and loss trials. These findings indicate that participants evaluate mixed trials in a similar way as loss trials. In other words: as soon as the lottery involves a loss, curiosity is decreased compared with gain trials.

**References**

1. Bürkner P-C. brms: an R package for bayesian multilevel models using Stan. J Stat Softw [Internet]. 2017;80(1):1–28. Available from: http://www.jstatsoft.org/v80/i01/

2. Barr DJ, Levy R, Scheepers C, Tily HJ. Random effects structure for confirmatory hypothesis testing: Keep it maximal. J Mem Lang. 2013;68(3):1–43.

3. Barr DJ. Random effects structure for testing interactions in linear mixed-effects models. Front Psychol. 2013;4:1–2.
