# Supplementary material for: Curiosity or savouring? Information seeking is modulated by both uncertainty and valence
Source: PLoS One. 2021 Sep 24;16(9):e0257011. doi: 10.1371/journal.pone.0257011 (PMC8462690; doi:10.1371/journal.pone.0257011)
Supplement: S3 Text — (DOCX) [file pone.0257011.s003.docx]

**S3 Text: Effects of uncertainty: entropy or absolute difference?**

The results of all experiments reported in the main text, show that outcome uncertainty is a strong driver of curiosity (see also Fig 2 and Fig 3). However, since outcome uncertainty is a combination of entropy and absolute difference between the monetary values (Equation 1), we investigated whether entropy and absolute difference both contribute significantly to curiosity/willingness to wait.

**Statistical analyses and model comparison**

In order to do so, we remodeled the data of Experiment 1A (as preregistered) and Experiment 2 (the willingness to wait experiment). Specifically we compared (i) model 1: with absolute difference, entropy, outcome valence (gain/loss) and absolute expected value as within-subject factors, (ii) model 2: with absolute difference, outcome valence (gain/loss) and absolute expected value as within-subject factors, and (iii) model 3: with entropy, outcome valence (gain/loss) and absolute expected value as within-subject factors.

As preregistered, we modeled the data of Experiment 1A using the clmm function of the ordinal package (Christensen, 2015) in R (R Core Team, 2013; RRID:SCR_001905). Models included all main effects described above and contained a full random effects structure (1,2). First, we assessed the significance of the effects of absolute difference and entropy as modeled in model 1 using the summary command. Next, we performed model comparisons in which we compared the Akaike Information Criterion (AIC) of the models using the anova command.

For Experiment 2 we modeled the data using the brm function of the BRMS package (3) in R (R Core Team, 2013; RRID:SCR_001905). Models included all main effects described above and contained a full random effects structure (1,2). All other conventions regarding the modeling are as for the primary statistical analyses (see *main text – 2. Methods – 2.4* *Experimental Design & Primary Statistical Analyses*). First, we assessed the significance of the effects of absolute difference and entropy as modeled in model 1. These coefficients were deemed statistically significant if the associated 95% posterior credible intervals were non-overlapping with zero. Next, we performed model comparisons using the loo method of the loo package (4) for approximate leave-one-out cross validation (LOO) using (Pareto-smoothed) importance sampling (PSIS). The LOO Information Criterion (LOOIC) of the models are reported here, since they have the same purpose as the AIC, which are used for the model comparisons of Experiment 1. LOOIC is intended to estimate the expected log predictive density (ELPD) for a dataset. The ELPD is based on the sum of the logs of the leave-one-out predictive density given the data without one of the data points (one of the trials). The difference in ELPD between the models gives an indication of how well the models explain the data.

**Effects of uncertainty: entropy or absolute difference?**

For Experiment 1A, we found that curiosity increased with both the absolute difference between the monetary values (**Ordinal:** Estimate = .78, SD = .097, z = 8.00, *p* = 1.24e-15) as well as with entropy (**Ordinal:** Estimate = .67, SD = .23, z = 2.99, *p* = 2.27e-3). This was confirmed by means of the model comparisons showing that the model containing absolute difference as well as entropy (model 1; AIC = 8139.2) explained significantly more variance in the curiosity responses than the model containing absolute difference (model 2; AIC =9533.6; LR.stat(6) = 1406.4, *p* = 2.2e-16) and the model containing entropy (model 3; AIC =8824.8; LR.stat(6) = 697.5, *p* = 2.2e-16). This suggests that a combination of entropy and absolute difference provides a better explanation of the data in Experiment 1A than entropy or absolute difference alone.

For Experiment 2, however, we found that willingness to wait increased with the absolute difference between the monetary values **(BRMS:** 95% CI [1.14,2.13]), but not with entropy (**BRMS:** 95% CI [-.69,.12]). The model comparisons showed that the model containing absolute difference as well as entropy (model 1; LOOIC = 5893.6, SE = 98.1) gives a better explanation of the data than model 3 that only includes entropy (LOOIC = 8342.5, SE = 69.7; ELDP_diff = -1224.4, SE_diff = 42.5). Model 1 also provides a better explanation of the data than model 2 that only contains absolute difference (LOOIC = 6390.2, SE = 94.8), but to a lesser extent given the smaller difference in expected predictive accuracy (ELDP_diff = -248.2, SE_diff = 20). All in all, this indicates that whereas the model including absolute difference as well as entropy provides the best explanation of the willingness to wait data. Still it should be noted that the modulation of willingness to wait is mostly explained by the absolute difference between the monetary values and to a lesser degree by entropy.

**Conclusion**

Overall, the results of the model comparisons show that models including both absolute difference as well as entropy provide a better explanation of the data than models including only one of these factors.

**References**

1. Barr DJ. Random effects structure for testing interactions in linear mixed-effects models. Front Psychol. 2013;4:1–2.

2. Barr DJ, Levy R, Scheepers C, Tily HJ. Random effects structure for confirmatory hypothesis testing: Keep it maximal. J Mem Lang. 2013;68(3):1–43.

3. Bürkner P-C. brms: an R package for bayesian multilevel models using Stan. J Stat Softw [Internet]. 2017;80(1):1–28. Available from: http://www.jstatsoft.org/v80/i01/

4. Vehtari A, Gabry J, Yao Y, Gelman A. loo: Efficient leave-one-out cross-validation and WAIC for Bayesian models. R Packag version 210 [Internet]. 2019; Available from: https://cran.r-project.org/package=loo
