# Supplementary material for: Curiosity or savouring? Information seeking is modulated by both uncertainty and valence
Source: PLoS One. 2021 Sep 24;16(9):e0257011. doi: 10.1371/journal.pone.0257011 (PMC8462690; doi:10.1371/journal.pone.0257011)
Supplement: S1 Table — (DOCX) [file pone.0257011.s004.docx]

**S1 Table: Overview of Experiments 1A, 1B, 1C and 2**

| **Overview of results Experiment 1A** | | | | | |
| --- | --- | --- | --- | --- | --- |
|  | **95% CI**  **BRMS** | **p-value  RM ANOVA** | **Effect size**  **RM ANOVA** | **BF_incl_ RM ANOVA** | |
| **Outcome valence (gain vs. loss)** | [.18,.51] | *p* = 2.93e-4 | η_p_^2^=.33 | BF = 1.27e+6 | |
| **Outcome uncertainty** | [.58,1.11] | *p* = 3.07e-8 | η_p_^2^=.61 | BF = 1.54e+20 | |
| **Expected value (absolute)** | [-.02,.26] | *p* = .13 | η_p_^2^=.068 | BF = .44 | |
| **Outcome valence * Outcome uncertainty** | [-.05,.09] | *p* = .20 | η_p_^2^=.049 | BF = .21 | |
| **Outcome valence * Expected value (abs)** | [-.09,.12] | *p* = .54 | η_p_^2^=.011 | BF = .20 | |
|  | | | | | |
| **Overview of results Experiment 1B** | | | | | |
|  | **95% CI**  **BRMS** | **p-value  RM ANOVA** | **Effect size**  **RM ANOVA** | | **BF_incl_ RM ANOVA** |
| **Outcome valence (gain vs. loss)** | [.61,1.86] | *p* = 9.69e-5 | η_p_^2^=.38 | | BF = 3.07e+15 |
| **Outcome uncertainty** | [1.17,2.60] | *p* = 1.22e-8 | η_p_^2^=.64 | | BF = 1.94e+19 |
| **Expected value (absolute)** | [.22,.47] | *p* = 2.42e-6 | η_p_^2^=.51 | | BF = 1.59 |
| **Outcome valence * Outcome uncertainty** | [.08,.37] | *p* = .066 | η_p_^2^=.10 | | BF = .21 |
| **Outcome valence * Expected value (abs)** | [.15,.51] | *p* = .001 | η_p_^2^=.28 | | BF = .51 |
|  | | | | | |
| **Overview of results Experiment 1C** | | | | | |
|  | **95% CI**  **BRMS** | **p-value  RM ANOVA** | **Effect size**  **RM ANOVA** | | **BF_incl_ RM ANOVA** |
| **Outcome valence (gain vs. loss)** | [.25,.71] | *p* = 2.75e-4 | η_p_^2^=.34 | | BF = 5.69e+7 |
| **Outcome uncertainty** | [1.56,2.94] | *p* = 4.80e-13 | η_p_^2^=.81 | | BF = 5.43e+48 |
| **Expected value (absolute)** | [.26.55] | *p* = 1.39e-5 | η_p_^2^=.45 | | BF = 862.7 |
| **Outcome valence * Outcome uncertainty** | [-.05,.16] | *p* = .995 | η_p_^2^=1.27e-6 | | BF = .20 |
| **Outcome valence * Expected value (abs)** | [-.02,.15] | *p* = .12 | η_p_^2^=.074 | | BF = .23 |
|  | | | | | |
| **Overview of results Experiment 2** | | | | | |
|  | **95% CI**  **BRMS** | **p-value  RM ANOVA** | **Effect size**  **RM ANOVA** | **BF_incl_ RM ANOVA** | |
| **Outcome valence (gain vs. loss)** | [.25,.98] | *p* = 1.46e-4 | η_p_^2^=.36 | BF = 3.27e+8 | |
| **Outcome uncertainty** | [1.23,2.33] | *p* = 3.38e-9 | η_p_^2^=.66 | BF = 5.32e+31 | |
| **Expected value (absolute)** | [-.06,.12] | *p* = .96 | η_p_^2^=7.85e-5 | BF = .14 | |
| **Outcome valence * Outcome uncertainty** | [-.01,.43] | *p* = .099 | η_p_^2^=.080 | BF = .24 | |
| **Outcome valence * Expected value (abs)** | [.08,.27] | *p* = 3.36e-4 | η_p_^2^=.33 | BF = 1.07 | |
