# Supplementary material for: Curiosity or savouring? Information seeking is modulated by both uncertainty and valence
Source: PLoS One. 2021 Sep 24;16(9):e0257011. doi: 10.1371/journal.pone.0257011 (PMC8462690; doi:10.1371/journal.pone.0257011)
Supplement: S1 Fig — (DOCX) [file pone.0257011.s005.docx]

**S1 Fig. Behavioural results of Experiment 1A, including the mixed condition.**

**
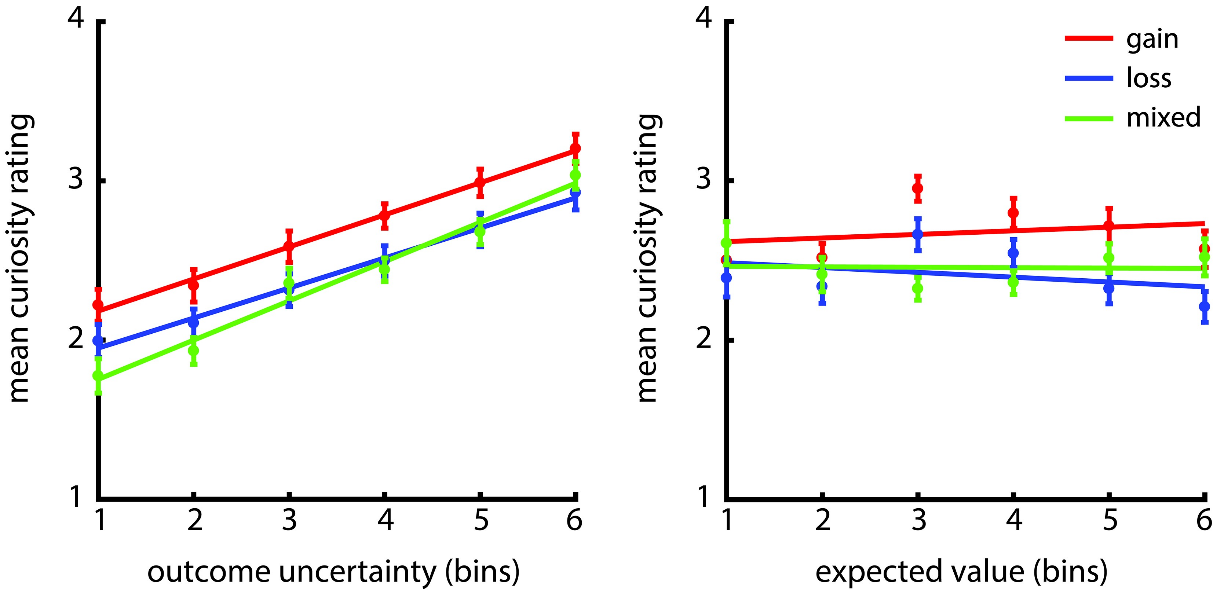
**

**S2 Fig. Behavioural results of Experiment 1A, including the mixed condition.**

The x-axes depict percentile bins of the values of outcome uncertainty (left) and of the standardized values of expected value (right). The y-axes depict the mean curiosity ratings for each percentile of outcome uncertainty and expected value for the gain trials (red), loss trials (blue) and mixed trials (green) separately. The results of Experiment 1A showed that curiosity was higher for gain trials than for mixed trials and loss trials. However, there was no difference between the mixed trials and loss trials. Curiosity increased monotonically with outcome uncertainty in all three conditions, but there was no significant modulation of curiosity by expected value. However, there was an interaction between outcome uncertainty and outcome valence, due to greater effects of outcome uncertainty for the mixed trials compared with the gain and loss trials.
